# Revolutionizing Alzheimer's Care with Wearable Technology to Enhance Quality of Life: A Systematic Review

**DOI:** 10.1002/alz70858_101645

**Published:** 2025-12-25

**Authors:** Nada Dahroug, Esra Ahmed Ibrahim Eltayeb, Amal Alamin, Esra Abdalla, Sabry Babiker Hassan Sayed, Mohamed Nasser Elshabrawi, Nadir G Abdelrahman

**Affiliations:** ^1^ Michigan State University, East Lansing, MI, USA; ^2^ Health Sciences Centre, Winnipeg, MB, Canada; ^3^ Port Said University, Port Said, Egypt

## Abstract

**Background:**

Alzheimer's disease is marked by progressive cognitive decline, including impairments in spatial awareness and short‐term memory. With the global prevalence of dementia, including Alzheimer's disease, expected to rise from 57 million cases in 2019 to nearly 153 million by 2050, innovative solutions are urgently needed. This systematic review aims to explore the role of wearable technology in improving the daily lives of Alzheimer's patients by evaluating its effectiveness.

**Method:**

Utilizing the Preferred Reporting Items for Systematic Reviews and Meta‐Analyses (PRISMA) guidelines, we conducted a systematic review on the efficacy of wearable technology in enhancing the quality of life for Alzheimer's patients. A comprehensive search of PubMed, Google Scholar, Cochrane, and Scopus was performed using keywords such as “assistive technology,” “wearable device,” and “Alzheimer's.” We included peer‐reviewed studies, published in English between 2015 and 2025, involving human participants diagnosed with Alzheimer's that assessed the impact of wearable devices on quality of life. Data extraction was independently conducted by three reviewers, and quality assessment was completed using the Critical Appraisal Skills Programme (CASP).

**Result:**

Out of 13,077 studies, five met the inclusion criteria, with the majority excluded due to lack of relevant outcomes. Qualitative analysis revealed that three studies using SenseCam, a wearable camera employed as a memory aid, demonstrated consistently significant improvements in autobiographical, episodic, and semantic memory over both short‐ and long‐term follow‐ups, along with short‐term reductions in depressive symptoms and enhancements in activities of daily living. A study with a Bluetooth earpiece that provided task reminders, instructions, and encouragement showed task initiation and completion rates increasing from below 35% to over 90%, indicating improved independence. On the other hand, the Microsoft HoloLens, an augmented reality headset, showed no significant improvements in task performance.

**Conclusion:**

This review identified wearable devices as promising tools for enhancing cognitive function, emotional well‐being, and daily activities in Alzheimer's care. While some devices significantly improved patients' quality of life, others require further refinement. These findings highlight the transformative potential of wearable technology and emphasize the need for continued research and user‐centered design to overcome implementation challenges and optimize integration in both clinical and home settings.

